# State-Aware Deep Item Response Theory using student facial features

**DOI:** 10.3389/frai.2023.1324279

**Published:** 2024-01-04

**Authors:** Yan Zhou, Kenji Suzuki, Shiro Kumano

**Affiliations:** ^1^Artificial Intelligence Laboratory, University of Tsukuba, Tsukuba, Japan; ^2^NTT Communication Science Laboratories, Nippon Telegraph and Telephone Corporation, Atsugi, Japan

**Keywords:** affective computing, multimodal learning, facial expression recognition, intelligent tutoring system, e-learning, Item Response Theory, learning analytics, educational data mining

## Abstract

This paper introduces a novel approach to Item Response Theory (IRT) by incorporating deep learning to analyze student facial expressions to enhance the prediction and understanding of student responses to test items. This research is based on the assertion that students' facial expressions offer crucial insights into their cognitive and affective states during testing, subsequently influencing their item responses. The proposed State-Aware Deep Item Response Theory (SAD-IRT) model introduces a new parameter, the student state parameter, which can be viewed as a relative subjective difficulty parameter. It is latent-regressed from students' facial features while solving test items using state-of-the-art deep learning techniques. In an experiment with 20 students, SAD-IRT boosted prediction performance in students' responses compared to prior models without the student state parameter, including standard IRT and its deep neural network implementation, while maintaining consistent predictions of student ability and item difficulty parameters. The research further illustrates the model's early prediction ability in predicting the student's response result before the student answered. This study holds substantial implications for educational assessment, laying the groundwork for more personalized and effective learning and assessment strategies that consider students' emotional and cognitive states.

## 1 Introduction

E-learning has become widely used in classrooms and has played a crucial role in today's learning, particularly with the rise of Massive Open Online Courses (MOOCs) and on-demand learning (Aboagye et al., 2021; Maatuk et al., 2022), especially in response to the COVID-19 pandemic. One prominent type of e-learning is Intelligent Tutoring Systems (ITS), which aims to provide learners with immediate and personalized instruction or feedback.

ITS uses an approach from the Vygotskian, i.e., Zone of Proximal Development theory (Reber, 1995), to provide each student with tasks that are of appropriate difficulty. According to this theory, students develop their skills and knowledge in situations where tasks cannot be solved by the student alone but can be solved with the help of an expert, known as scaffolding. Inappropriate task difficulty can lead to minimal progress and result in negative affect such as boredom or frustration (Fenza et al., 2017). Literature on ITS has repeatedly reported the complex relationship between a student's ability and affect. Pekrun (2011) discussed the reciprocal causation between affect and learning development and acknowledged the importance of affect for learning. Therefore, the student model should predict student performance by modeling both student affective states and abilities, as they are reciprocally complementary in understanding students.

Regarding ability modeling, Item Response Theory (IRT) has primarily been used to estimate students' abilities and item difficulties based on their answer responses. IRT is based on the local independence assumption that the probability of a person correctly answering a target item does not depend on its response to any other items. In recent years, there have been attempts to combine deep neural networks with IRT to improve prediction performance on responses (Cheng et al., 2019; Uto and Uchida, 2020; Tsutsumi et al., 2021; Zhou et al., 2021). The Deep Item Response Theory (Deep-IRT) model (Tsutsumi et al., 2021) has shown better response prediction performance than standard IRT models. However, IRT assumes that student and item parameters are static and do not account for student state changes.

A number of emerging studies predicted students learning performance by estimating learning engagement and examining its relationship with student performance. Due to the affect-rich nature of facial expressions, emotion recognition methods have often been used for predicting learning engagement (Joshi et al., 2019; Abedi and Khan, 2021; Ruiz et al., 2021). Positive emotions were referred to as emotional engagement in some studies (Liu S. et al., 2022). Facial-video-based learning engagement recognition studies also have the potential to predict student performance. For example, Joshi et al. (2019) predicted learning engagement from facial videos in different classes, such as success on the first attempt and giving up, indicating whether the student correctly answered the question.

To handle student ability and states simultaneously for more accurate student performance prediction, we propose a novel deep learning-based student state-aware IRT model that uses students' answers and facial videos. The student state parameter, the key parameter of our model, is estimated using facial videos taken while the student is solving problems. The estimated student state parameter is then incorporated into the logistic function of Deep-IRT model to explain a remaining factor that (Deep-)IRT models do not handle. A human experiment was conducted to collect response logs and facial videos, and cross-validation was performed to evaluate the proposed model. The proposed model achieved the best student response prediction performance compared to previous methods that ignored student ability or student state. Additionally, the proposed model demonstrated its interpretability of student response prediction by accurately estimating IRT parameters, including student ability and item difficulty, and interpreting the student state as a relative subjective difficulty parameter. The good interpretability of the proposed model can be used as feedback to students and other stakeholders, and it is also important for ITS to provide prompt intervention in learning.

## 2 Related works

In this section, we first introduce recent developments in facial-video-based methods for learning engagement prediction. We then discuss recent works of log-based methods on response data mining, including IRT and Knowledge Tracing, and how previous works handled student states. Finally, we propose a unified model using facial-video-based and log-based methods to improve student response prediction performance.

There are primarily two trends in recent studies to improve the accuracy of the learning engagement prediction task. One is to use a more accurate pre-trained facial expression recognition model to extract robust facial features against face occlusion and variation. The other is to use a sequential modeling method, such as RNN, to learn temporal features among frames. Among the facial features used, action units belong to an encoding system (Ekman and Friesen, 1978) that comprehensively and anatomically describes the actions of individual muscles or groups of muscles for facial movements, such as upper lip raiser and chin raiser. Action units have been widely used for high-order facial analysis processes, such as facial expression recognition. Joshi et al. (2019) utilized OpenFace to extract action units, head pose, and eye gaze features from videos and then employed a two-linear layer-based neural network model to predict learning engagement. The model was trained on the summary statistic features of action units, which are commonly used and easy to implement in learning engagement prediction, although the summary statistics of OpenFace facial features lost much information. Following the work of Ruiz et al. (2021) utilized a deep recurrent model (LSTM) to learn intermediate facial features from a convolutional neural network-based facial expression recognition model in the engagement prediction task. Similarly, Abedi and Khan (2021) employed another deep learning model, Temporal Convolutional Network (TCN, Bai et al., 2018), to learn facial video features extracted by a pre-trained CNN-based facial expression recognition model.

However, the relationship between facial features and learning engagement is usually complex and context-dependent (Barrett et al., 2019; Durán and Fernández-Dols, 2021; Witkower et al., 2023). Consequently, the internal state of students cannot be wholly predicted from facial features alone. Exploring other emotion measures used in learning domain studies could be intriguing. For instance, hand-over-face gestures (Behera et al., 2020), head and eye movements (Zhan et al., 2016; Behera et al., 2020) as indicators of increasing (subjective) item difficulties, and electrodermal activity (EDA) as an indicator of children's learning engagement (Park et al., 2019), could provide valuable insights. Discussion text data from MOOCs were also used to understand learning engagement and its relationship with student performance: Liu Z. et al. (2022) investigated how cognitive presence, related to critical thinking and high-order knowledge acquisition and application, changes according to different course discussion pacing and discussed their relationships on student performance; Liu S. et al. (2022) developed a text classification model to analyze the course discussion so it can recognize emotional and cognitive engagement and then jointly predict student performance. However, these considerations are beyond the scope of this paper.

In addition to IRT for estimating student ability from student response logs, another paradigm for mining log data is Knowledge Tracing (KT), which interprets the responses from a learning perspective. KT assumes that new skills are acquired while solving a sequence of questions. In KT, the probability of a correct response depends on a latent variable, the student's knowledge state, which is learned by a Hidden Markov Model using students' answers. The knowledge state changes with each question solved, indicating whether or not the skill has been mastered, simulating the knowledge acquisition process. The probability of a correct response depends on the knowledge states. Unlike IRT, KT usually does not assume inter-differences among students or question items, i.e., the ability and the difficulty. Some works have synthesized these two model paradigms to complement each other's limitations and improve the accuracy of predicting student performance, such as Khajah et al. (2014) and Corrigan et al. (2015). In some instances, the combination of these models has resulted in enhanced student performance prediction. Ghosh et al. (2020) used attention-based deep neural network models for KT and integrated IRT to capture individual differences. It reached a state-of-the-art response prediction performance compared to previous KT models. However, since the model does not have interpretable parameters, it is hard to know how the student's ability changes during the learning and thus is limited to further applications in education. For model interpretability, we did not use such an attention-based method; instead, we imposed restrictions on the neural network model to ensure parameter interpretability.

Studies have used additional features to estimate latent variables in IRT or KT, resulting in improved student learning performance prediction. Usually, these additional features include items' content information or facial features related to student forgetting behaviors. For example, Zhou et al. (2021) used images as items and trained a Convolutional Neural Network regressor to estimate the item parameter β. This approach demonstrated improved prediction performance compared to methods that used conventional latent regressors to estimate item parameters from handcrafted facial features. González-Brenes et al. (2014) proposed the Feature Aware Student Knowledge Tracing Model, a general framework for using additional features to estimate knowledge state variables or response probability directly. Large Language Models (LLMs) are potential tools for analyzing item texts. Many studies have used LLMs such as ChatGPT in the education scenario, including generalizing questions and hints (Bohacek, 2023), playing as a learning tutor chatbot (Yadav et al., 2023), and even scoring essays (Caines et al., 2023). With item texts, it is possible to use LLMs to estimate item characteristics and cluster items for different skills, which can save expert annotations and accelerate large-scale applications in the future. However, in this study, we focus on modeling student states using facial features, and thus, the extension of using LLMs in student performance prediction is out of scope.

Unlike facial-video-based methods, log-based methods typically do not assume that the student's affective state influences the response. However, the prediction performance may decrease in some situations, such as when high-level students fail due to boredom or anxiety. Some works have regarded it as the affect or engagement factor in log-based methods. Johns and Woolf (2006) was the first to consider the student affective state by combining IRT and KT. Using a Hidden Markov Model to recognize engagement/disengagement using response time, they integrated the recognition result with IRT. The engagement factor was similar to the knowledge state variable in KT. If the student was engaged, the response was predicted using IRT. Otherwise, the correct response probability would be considered as guessing behavior, resulting in a low probability. However, they did not find significant improvement, but they proposed a feasible approach to handle disengaged responses in IRT by excluding them from the ability estimation process. Subsequently, another study (Corrigan et al., 2015) continued this work and incorporated a Hidden Markov Model to learn the student affective state into the Bayesian Knowledge Tracing model. The results did not show overall improvement, but it outperformed in cases of guessing and slip (i.e., failure of a task despite possessing the necessary knowledge), with higher probabilities observed in low engagement. After the initial development of the engagement-aware IRT model, several subsequent studies utilized response time to detect engagement in various IRT models, primarily in computer adaptive testing (Wang and Xu, 2015; Nagy and Ulitzsch, 2022). The combination of IRT and KT is promising to handle inter-differences among students, question items, and dynamic changes in student state. However, in this study, unlike the process of knowledge acquisition by solving a sequence of questions, the student's affective state changes more frequently, and the student may experience a series of different affect within solving one question.

Integrating facial-video-based methods with Deep-IRT can potentially improve student response prediction performance. Although response logs are easy to collect, they may have little information about the student's state compared to facial videos. Sharma and Giannakos (2020) suggested that ubiquitous and noninvasive data such as facial videos and logs should be used together for future multimodal data learning analytics. D'Mello et al. (2007) highlighted the need for a multimodal fusion model to detect student affect, as the limitations of any single modality can be overcome by using multiple modalities to complement each other and improve overall prediction performance. On the other hand, some works have already used facial-video-based and log-based methods in ITS to model student performance by estimating student affect and ability. For example, Park et al. (2019) developed a robot to assist children's language learning. The robot estimates learning engagement by using the child's facial expression and electrodermal activity. It also estimates literacy skills by analyzing the child's historical answers. Based on the estimations of engagement and literacy skills, the robot provides suitable learning content by updating the agent's policy instead of predicting student performance.

However, few studies considered using facial features and response data together to understand learning engagement and predict student performance. The response data are useful as they reflect idiosyncratic characteristics of participants and items, e.g., abilities and difficulties, making the student performance prediction more accurate. In this study, we propose a unified method to model student ability and state for higher student response prediction performance. By introducing a student state latent variable into IRT and estimating it using facial features, we can leverage the advantages of both facial-video-based methods and log-based methods to improve prediction performance. Even though there exist many studies that tackled emotion/engagement recognition in the education domain, the proposed method is the first unified method to use facial features and response data. We believe our study can contribute to the current discussions on learning engagement estimation and student performance prediction.

## 3 Proposed method

To enhance the performance of student response prediction, we incorporate a facial-video-based deep student state regressor into Deep-IRT model. Our approach utilizes facial videos to latently regress the student state parameter, designed to explain the residuals unaccounted by static person ability and item difficulty parameters in conventional item-response theory. This section will first introduce the standard IRT and Deep-IRT models. Then, we will explain how to integrate the facial-video-based deep student state regressor into Deep-IRT model to estimate the student state using facial videos.

### 3.1 Item Response Theory and its implementation in deep learning

We will introduce the One-Parameter Logistic (1PL) Model, the simplest form of Item Response Theory (IRT). In this model, the response of student *j* to item *i* is denoted as *y*_*ij*_∈{0, 1}, where 0 indicates an incorrect response and 1 indicates a correct response. The probability of student *j* answering item *i* correctly can be defined as:


(1) $$\begin{array}{l} P\left(y_{ij}=1|\theta_{j},\beta_{i}\right)=\sigma \left(\theta_{j}-\beta_{i}\right)\\ \;=\frac{1}{1+\exp\left(-\left(\theta_{j}-\beta_{i}\right)\right)} \end{array}$$

Here, σ represents the sigmoid function, a monotonically increasing function that outputs values between 0 and 1. The parameter θ_*j*_ represents the ability of student *j*, while β_*i*_ represents the difficulty of item *i*. Higher values of θ_*j*_ or β_*i*_ indicate a more proficient student or a more difficult item. According to the probability function of the 1PL model, if an item is more difficult (i.e., β_*i*_ is larger), the probability of a correct response decreases. Conversely, if a student is more proficient (i.e., θ_*j*_ is larger), the probability of a correct response increases. In addition, 1−*P*(*y*_*ij*_ = 1|θ_*j*_, β_*i*_) represents the probability of an incorrect response.

Deep-IRT model (Tsutsumi et al., 2021) implements the 1PL model in deep neural networks for parameter estimation. In their model, ability parameter θ and difficulty parameter β are latent-regressed using two independent deep IRT regressors, namely student network $F_{\theta }$ and item network $F_{\beta }$. The input to these networks is a one-hot vector representing the index of the student or item. The length of the one-hot vector depends on the number of students or items in the dataset. For example, if there are five students, the one-hot vector for the fifth student will be [0, 0, 0, 0, 1]. With the input of the student index vector, the student network outputs the estimated student ability parameter θ_5_ for the fifth student. A similar process is applied to estimate the item parameter using item network $F_{\beta }$. Both networks consist of several linear layers with a hyperbolic tangent activation function. Finally, the estimated item response parameters θ_*j*_ and β_*i*_ are used to predict the probability of student *j* giving a correct response to question item *i* using the sigmoid function, as in Equation 1. The predicted response is 1 if *P*(*y*_*ij*_ = 1|θ_*j*_, β_*i*_)≥0.5 and 0 otherwise. The original Deep-IRT model outputs the predicted probabilities for each class, i.e., the correct and incorrect response. However, in this study, we removed the last linear layer and the following softmax function in the original Deep-IRT model because they made the interpretation of IRT parameters difficult. Therefore, the model only outputs the predicted probability of a correct response in the same way as the standard 1PL, i.e., *P*(*y*_*ij*_ = 1|θ_*j*_, β_*i*_).

Unlike traditional IRT models that estimate parameters using statistical inference methods such as maximum likelihood estimation, Deep-IRT utilizes independent Student/Item Networks to estimate item-response parameters by leveraging inter-student and inter-item correlations. The model parameters are estimated through gradient descent optimization, minimizing the cross-entropy loss between predicted and true responses.

### 3.2 The proposed model: State-Aware Deep Item Response Theory (SAD-IRT) for modeling student state in IRT with deep facial-video-based methods

**Figure 1:**
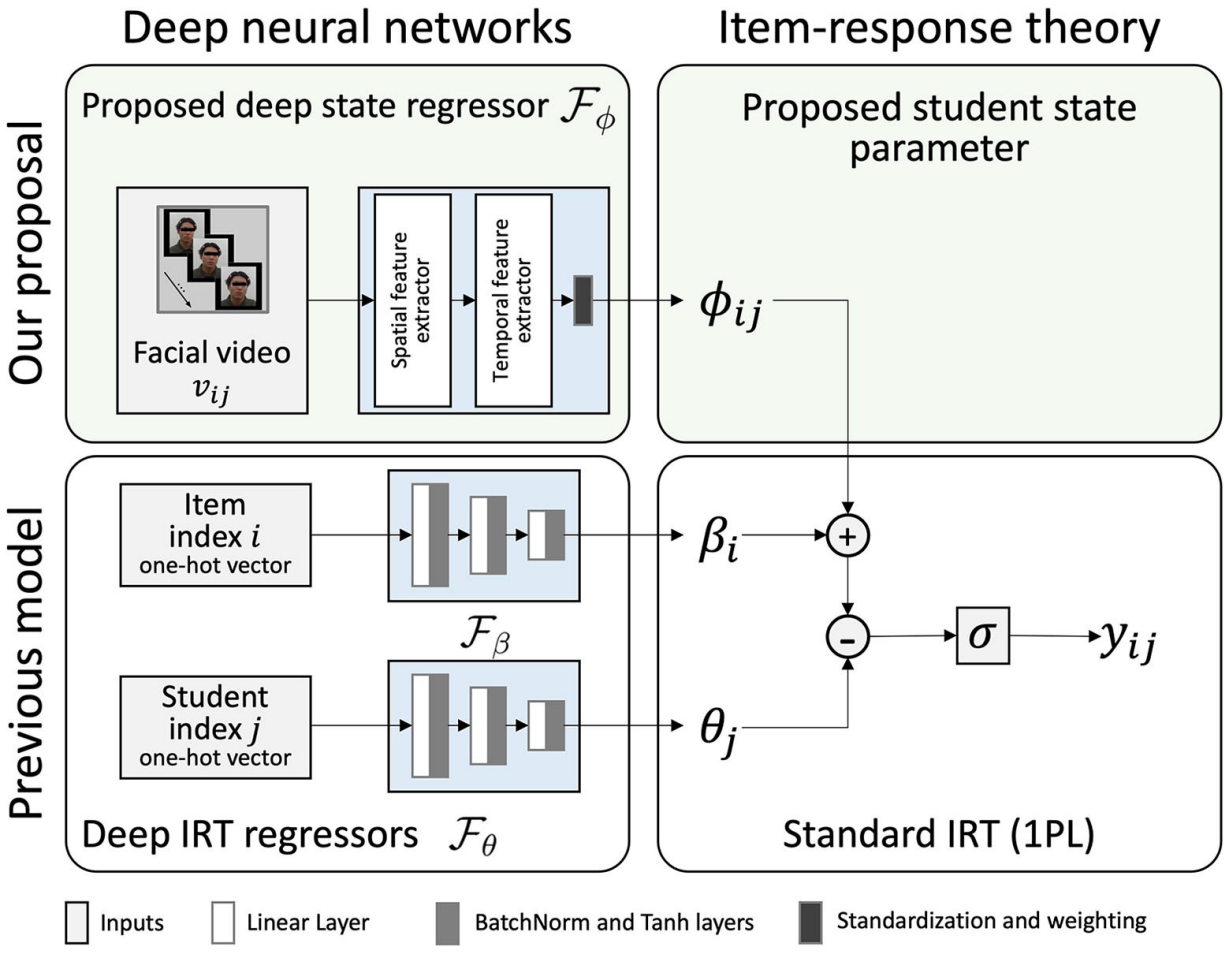
Proposed model: State-Aware Deep Item Response Theory (SAD-IRT) uses facial videos **(upper left)** to estimate the student state parameter **(upper right)** incorporated into Deep-IRT's logistic function to predict response. With the operations of standardization and weighting (i.e., the multiplication of the normalized estimation with a scalar weight parameter) in the deep state regressor, student state ϕ_*ij*_ estimations are constrained to be zero-mean and weighted variance.

In this study, we introduce a novel deep neural network model that estimates the student state using facial videos of each question-solving process in conjunction with Deep-IRT model. We call it State-Aware Deep Item Response Theory (SAD-IRT). As shown in Figure 1, SAD-IRT predicts the response of student *j* to item *i* by incorporating the newly proposed student state parameter, denoted as ϕ_*ij*_, into the logistic function of Deep-IRT:


(2) $$\begin{array}{ll} P\left(y_{ij}=1|\theta_{j},\beta_{i},\phi_{ij}\right) & =\sigma \left(\theta_{j}-\left(\beta_{i}+\phi_{ij}\right)\right) \end{array}$$

Here, ϕ_*ij*_ is assumed to follow a zero-mean normal distribution, allowing it to be interpreted as a relative subjective difficulty parameter.

In SAD-IRT, the proposed deep state regressor, denoted as $F_{\phi }$, estimates ϕ_*ij*_ as shown in Equation 3. The video *v*_*ij*_ collected during the student *j* solving problem *i* is input into the deep state regressor $F_{\phi }$. $F_{\phi }$ extracts facial features using deep neural network-based spatial and temporal facial feature extractors sequentially. The spatial feature extractor extracts a sequence of features from the input facial video by each frame or a sliding temporal window. Then the temporal feature extractor extracts the temporal feature and estimates the student state ϕ_*ij*_ from the spatial features. Here, the temporal feature extractor is similar to the Feature Aware Student Knowledge Tracing Model (González-Brenes et al., 2014), where the response result prediction was obtained through a weight vector multiplying a feature vector. However, instead of a weight vector parameter, we can use a deep sequential model such as TCN to learn spatial features as a time series input. By passing the output of the temporal feature extractor to a linear layer, we get its output as a scalar representation of the student state. As we assume ϕ_*ij*_ represents the relative subjective difficulty, we need its distribution to have a zero mean. Therefore, we estimate ϕ_*ij*_ by standardizing the scalar representations and multiplying them with a variance weight parameter, resulting in a zero mean and weighted variance distribution of the estimations of ϕ_*ij*_ as illustrated in Figure 1.


(3) $$\begin{array}{l} \phi_{ij}=F_{\phi }\left(v_{ij}\right) \end{array}$$

The model is trained using the steps outlined in Algorithm 1. As the input video duration varies, we apply zero padding to all extracted spatial facial features to ensure consistent sequence lengths. The model parameters are updated by minimizing the cross-entropy loss L, as shown in Equation 4.

**Algorithm 1:** Training Procedure for SAD-IRT Model.

Require: $F_{\phi },F_{\theta },F_{\beta },\mathrm{D}$
while not converged **do**
(*v*_*ij*_, *i, j, y*_*ij*_)←sample batch ⅅ ⊳ facial video *v*_*ij*_, item index *i*, student index *j* and response *y*_*ij*_
$\phi_{ij}\leftarrow F_{\phi }\left(v_{ij}\right)$ ⊳ Latent-regress student state ϕ_*ij*_ with facial features extracted from the video *v*_*ij*_ with spatial feature and temporal feature extractors
$\theta_{j}\leftarrow F_{\theta }\left(j\right)$ ⊳ Estimate student ability parameter θ_*j*_ from input index *j*
$\beta_{i}\leftarrow F_{\beta }\left(i\right)$ ⊳ Estimate item difficulty parameter β_*i*_ from input index *i*
*P*(*y*_*ij*_ = 1|θ_*j*_, β_*i*_, ϕ_*ij*_)←σ(θ_*j*_−(β_*i*_+ϕ_*ij*_)) ⊳ Probability of correct response
$\left\{\phi ,\theta ,\beta \right\}\leftarrow \nabla_{\left\{\phi ,\theta ,\beta \right\}}L\left(y_{ij},P\left(y_{ij}=1|\theta_{j},\beta_{i},\phi_{ij}\right)\right)$ ⊳ update parameters
end **while**


(4) $$\begin{array}{l} ℒ\left(y_{ij},P\left(y_{ij}=1|\theta_{j},\beta_{i},\phi_{ij}\right)\right)=\\ \sum_{i=1}^{I}\sum_{j=1}^{J}[\log\left(P\left(y_{ij}=1|\theta_{j},\beta_{i},\phi_{ij}\right)\right)y_{ij}\\ +\log\left(1-P\left(y_{ij}=1|\theta_{j},\beta_{i},\phi_{ij}\right)\right)\left(1-y_{ij}\right)] \end{array}$$

## 4 Experiment

This section explains the experiment to collect a dataset to evaluate the methods described in Section 5. The dataset comprises participants' answer logs and facial videos. In the Learning Analytics research community, several open datasets are available that contain face or log data, which are commonly used for various purposes. For example, the DAISEE dataset (Gupta et al., 2022) provides facial video data with annotations of learning engagement, while the student engagement dataset (Delgado et al., 2021) offers in-the-wild facial video data for learning engagement analysis. Additionally, the ASSISTment dataset^1^ provides response log data for educational assessment. However, to the best of our knowledge, no open dataset is available that includes both face and log data for learning purposes.

### 4.1 Participants

A total of 20 participants, Japanese native students at the university, were recruited for the experiment after providing informed consent. The inclusion criteria for participant selection were as follows: (1) aged between 18 and 30 years old, (2) no prior full-time work experience, and (3) good physical and mental health to use experimental devices and complete the main tasks.

### 4.2 Experiment apparatus

The experiment questions used in this study were adapted from recruitment assessment questions in Japan, specifically from the *Synthetic Personality Inventory* (SPI^2^). The goal of this experimental study was to target general abilities using tests that are frequently utilized both in research and practical settings. SPI fits this criterion, as it is a family of aptitude tests widely used in Japanese recruitment to assess fresh university graduates' personalities and capabilities, including verbal and numerical reasoning^3^. SPI has also been utilized in e-Learning studies with Japanese university students, as seen in Takegami (2010), Arai (2012), and is comparable to studies in English, such as those using MathTutor (Ruiz et al., 2021) or literacy education robots (Park et al., 2019). Given the experiment's context, conducted at a Japanese university with native Japanese students, we consider SPI an appropriate choice.

**Figure 2:**
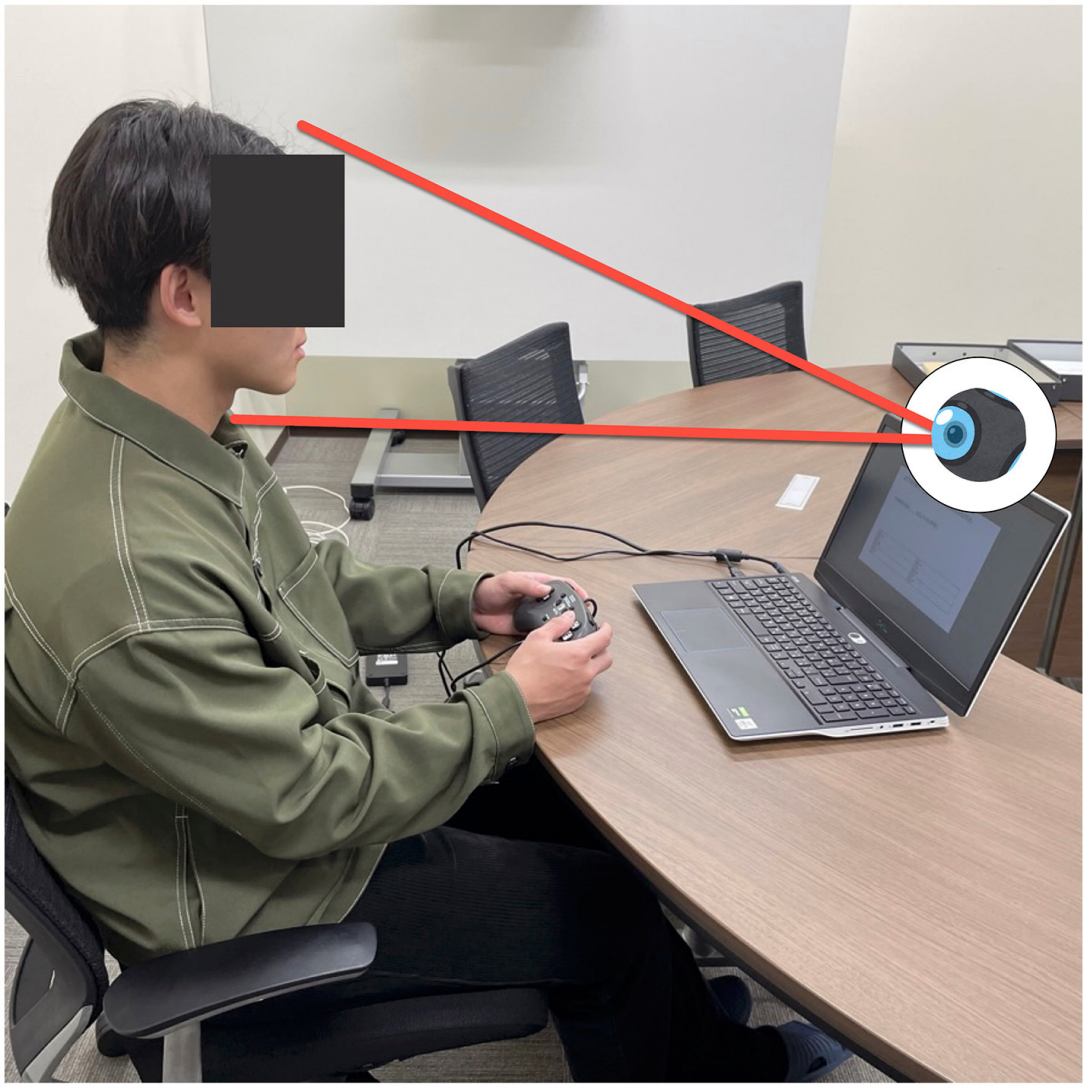
Experiment settings: the main experiment was conducted on a PC laptop, featuring an inbuilt camera for capturing facial videos and a game controller for recording participant responses to prevent potential face obstructions by hand-over-face behaviors.

Furthermore, math items in SPI often require respondents to use paper and pencil, which can hinder capturing their frontal face for facial analysis, as also reported in Behera et al. (2020). Therefore, to prioritize clear facial recording, we chose to use only literacy problems (including verbal reasoning, lexical skills, and reading comprehension questions) in our study. An example of such a literacy problem was provided in Figure 2. These questions used in the main experiment were excerpted from practice books^4^,^5^,^6^.

The experiment required each participant to solve a minimum of 50 questions, ensuring the reliability of the estimation results. It was necessary because the proposed model incorporates the IRT model, which typically requires a test size of at least 50 or more to obtain accurate estimations. Although we did not employ the standard IRT with statistical inference directly in this study, we still adhered to the suggestion from the IRT research community, considering it a rule of thumb.

**Figure 3:**
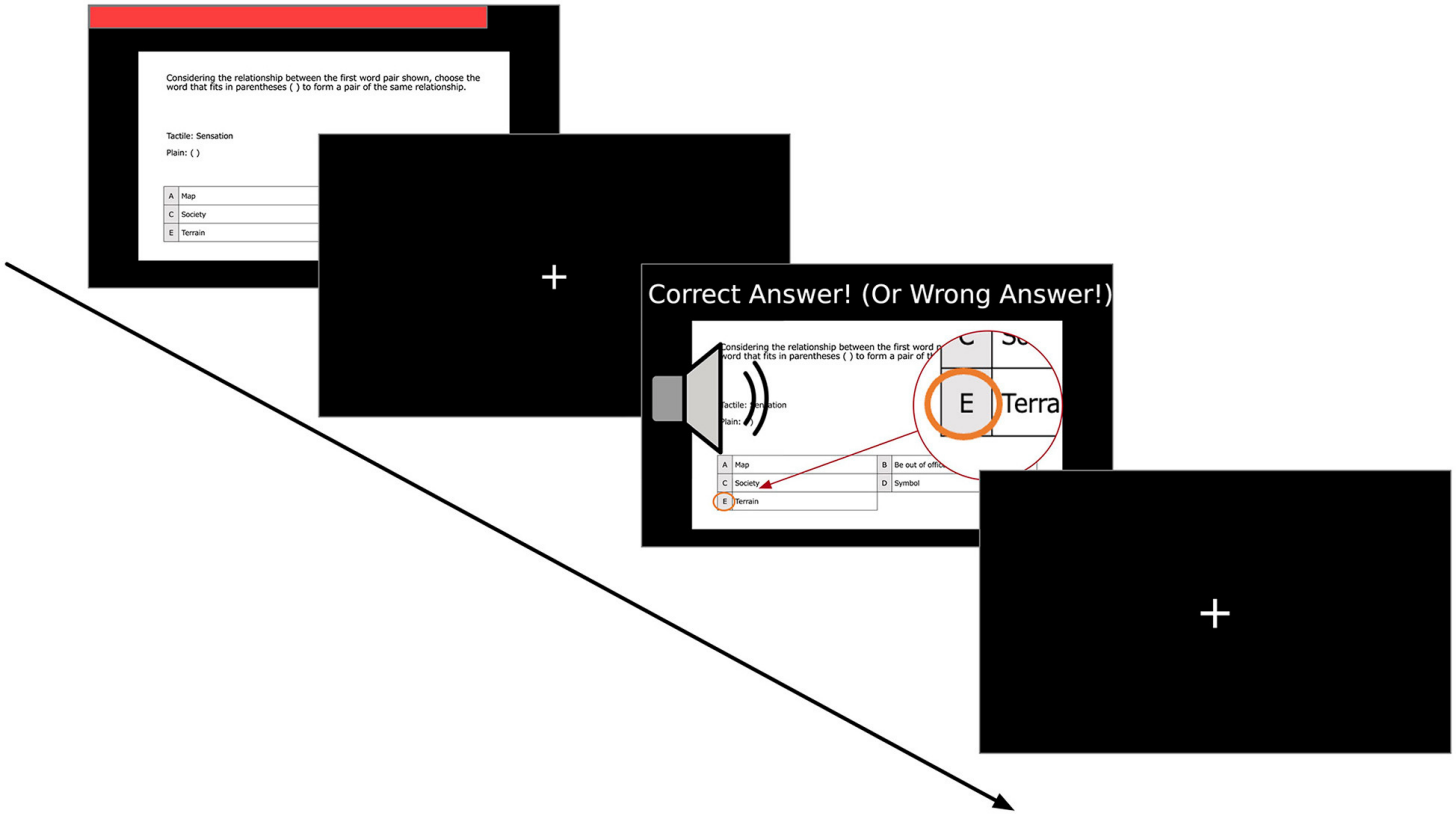
Timeline for each item in the main task. (1) Presentation of Problem (up to 60 s): problem displayed on the screen with a red time bar shown above; Time bar shrinks until it disappears at the time limit. (2) Fixation Cross (1 s): a brief pause before the feedback phase; Fixation Cross Presented at the center. (3) Feedback Display (2 s): text message informing whether the last answer was correct; A sound effect played differently depending on the response result; Correct choice highlighted to provide with visual reinforcement; (4) Fixation Cross (1 s): repeat showing fixation cross again and move on to the next.

The software development environment used for this study was Windows 11 (ver. 21H2) and PsychoPy (ver. 2022.1.2) (Peirce et al., 2019). PsychoPy is a Python-based Psychology Experiment Development Environment. We utilized the OpenCV (Bradski, 2000) interface (ver. 4.4.0.46) in the PsychoPy experiment program to enable video recording with the laptop's built-in camera. The experimental content was displayed on the laptop screen, as shown in Figure 2. The question was presented in the center of the screen, while a moving bar at the top of the screen indicated the remaining time, as depicted in Figure 3.

For participant interaction, a game controller with six buttons was used. Each button corresponds to one of the six choices in the experimental problem. The decision to use a game controller was based on its ergonomic design, which allowed for easy gripping with both hands. Throughout the experiment, participants held the game controller and responded to questions by pressing the appropriate buttons. The game controller was chosen over a keyboard due to its limited number of buttons and ease of finger movement. Additionally, using the game controller helped prevent participants from obstructing their faces with their hands, ensuring better video quality with fewer facial occlusions.

### 4.3 Procedure

The experiment consisted of two sessions: a practice session and a main session.

#### 4.3.1 Practice session

In the beginning, participants were provided with an explanation of the experiment, including the procedure, the operation of the experiment interface, the process of capturing facial videos during the experiment, and the use of recorded videos for research purposes. After obtaining informed consent from the participants, the practice session started.

During the practice session, participants completed two pre-tasks to familiarize themselves with the experiment procedures. The first practice task involved solving four practice problems with no time limit. In the second practice task, participants solved the remaining four problems within a maximum time limit of 60 seconds, replicating the setting of the main session and allowing participants to become accustomed to the time constraint.

#### 4.3.2 Main task

In the main session, participants answered 50 questions divided into two parts, as depicted in Figure 3. Each question allowed a maximum response time of 60 seconds. If the time limit was reached, the program automatically moved on to the next question. Facial videos and log data were recorded for each question, including question responses and response time. The main task was further divided into two parts, with a break in the middle. The first part consisted of 25 literacy problems, encompassing lexical knowledge and reading comprehension. These 25 problems were randomly selected from a total of 44 problems for each participant. After completing the first 25 questions, participants were given a rest period of up to 10 min. They had the flexibility to end the break at any time. The second part of the main task comprised 25 verbal reasoning problems. These 25 problems were shuffled before being presented to the participants. The experiment concluded once all the questions in both parts were answered.

Moreover, as the potential issue mentioned in Section 2 that facial expressions used as emotion indicators might be insufficient, we attempted to mitigate the issue by designing the experiment to minimize communicative emotional expressions (Chovil, 1997), with each participant solving problems alone in a room. Therefore, the context of the experiment was fixed. However, we acknowledge that this design was not flawless, as participants were aware that their faces were being recorded for ethics and compliance.

The experiment yielded 1,000 data samples (50 items · 20 respondents). After the experiment, the responses for each sample were encoded as 1 for a correct response and 0 for an incorrect response.

## 5 Evaluation

In this section, we evaluate the proposed method explained in Section 3.2 using a dataset collected.

### 5.1 Evaluation settings

We implemented our proposed model using PyTorch (ver. 1.12) and Python (ver. 3.97) and utilized an Nvidia RTX 3080 GPU for accelerated computation. The Adam optimizer was employed for training, with a learning rate of 1e-03, a batch size of 64, and a total of 200 epochs.

### 5.2 Implementations of proposed and baseline models

Regarding the implementation of the proposed SAD-IRT model, as shown in Figure 1, in our proposed model, we extract spatial features from the input video using a spatial feature extractor, which can be any facial-video-based model, such as facial expression recognition model, and we can use the middle-layer or penultimate-layer output as the high-level facial features. These features are obtained from facial videos *v*_*ij*_ recorded during the student's task-solving process. As highlighted in a survey paper on facial analysis (Li and Deng, 2020), one common challenge in facial analysis tasks is the lack of sufficient training data. To address this, using pre-trained models to extract features provides a solution to train facial-video-based models on smaller datasets. Regarding the spatial features, action units extracted with OpenFace have been commonly used as facial features in previous works such as Joshi et al. (2019), Ruiz et al. (2021), Kamath et al. (2022), Wang et al. (2023). At first, we implemented the facial-video-only (OpenFace) model proposed by Joshi et al. (2019). This model is a neural network with two linear layers. Each layer has 100 nodes and is followed by a ReLU activation function. The model learns summary statistics features extracted by OpenFace, including the maximum, minimum, mean, and SD values of action units, eye gaze, and head pose.

However, OpenFace primarily focuses on static images, which may not adequately capture video information across frames. In this study, we employed another pre-trained facial feature extractor called *Masked Autoencoder for facial video Representation LearnINg* (MARLIN) from Cai et al. (2023). MARLIN is a variational auto-encoder-based facial reconstructor that extracts spatial^7^ facial features from videos. Cai et al. (2023) reported good performance of MARLIN in downstream tasks such as in-the-wild facial expression recognition. Therefore, we utilized MARLIN model as spatial feature extractor in our study. In practice, MARLIN divided the input video into a sequence of video clips and extracted features from each video clip sequentially. In our dataset, the longest MARLIN feature length was 56, the shortest was 2, and the average length was 23.10 ± 14.45.

We then employed a deep sequential model, TCN, as the temporal feature extractor in $F_{\phi }$. TCN has been previously used for predicting learning engagement using the facial emotion recognition model's middle layer features from videos (Abedi and Khan, 2021). We use the penultimate layer output of the TCN module as the high-level facial video features. We pass these features through a linear layer with a batch normalization layer and hyperbolic tangent function to obtain a scalar representation. We then get the estimations of ϕ_*ij*_ after the operations of normalization and weighting for the scalar representations.

In SAD-IRT and Deep-IRT, we improved the prediction performance by combining student and item networks with additional linear and Batch Normalization layers. The Batch Normalization layer (Ioffe and Szegedy, 2015) standardizes layer inputs after weight updates, stabilizing the learning process and accelerating optimization, particularly in deeper networks.

Since the proposed model, SAD-IRT, combines the deep state regressor and Deep-IRT models, we compare it to the two separate baseline models: The facial-video-only independent deep state regressor (consisting of two feature extractors, i.e., MARLIN and TCN) and Deep-IRT models. We expect that SAD-IRT model will exhibit better prediction performance by integrating Deep-IRT with the proposed deep state regressor $F_{\phi }$.

**Figure 4:**
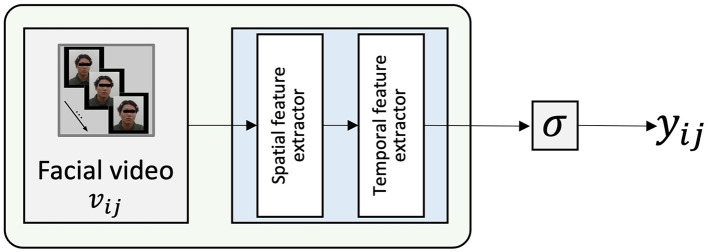
Facial-video-only (MARLIN-TCN) Model, the output classification scalar value before the sigmoid function corresponds to the student state ϕ_*ij*_ without standardization and weighting operation in Figure 1.

Facial-video-only (MARLIN-TCN) model (Figure 4) is a facial-video-based model used for response prediction. Similar to the facial videos processed in SAD-IRT, MARLIN-TCN model was trained with videos by using the MARLIN spatial and TCN temporal feature extractor. Unlike SAD-IRT, which uses deep state regressor to estimate the student state, MARLIN-TCN model makes predictions directly from its outputs and thus does not need the normalization and weighting operations to constrain outputs in the end.

**Figure 5:**
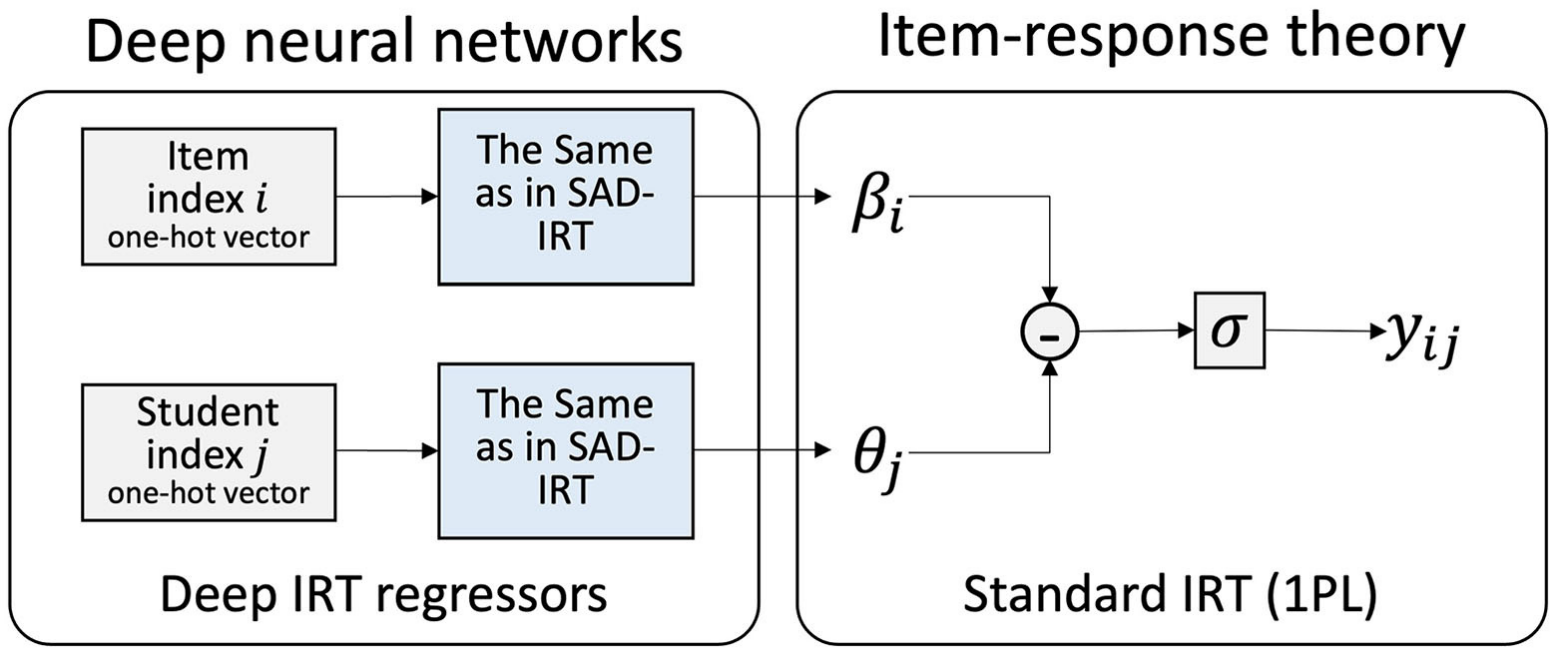
Baseline Deep-IRT model (Tsutsumi et al., 2021).

As for Deep-IRT baseline models, one is Deep-IRT model (as illustrated in Figure 5), which follows a similar structure and is also integrated with the proposed model. The other one is the original Deep-IRT w/o BatchNorm model in Tsutsumi et al. (2021), which has only two linear layers with every 50 nodes in the student/item network. There is only a hyperbolic tangent activation function after each linear layer, without a Batch Normalization layer. The two Deep-IRT models solely learn participants' answers for prediction. By providing student and item indices, Deep-IRT model estimates student and item parameters, θ and β, and then predicts whether the response is correct.

### 5.3 Evaluation methods

To evaluate the prediction performance of the proposed model and the baseline models, we employed a 20-fold stratified cross-validation. Since the dataset was imbalanced, with around 72% positive response samples, we used stratified cross-validation to ensure that the training and test sets had the same proportion of positive and negative samples as the entire dataset. It allowed us to obtain cross-validation results that were close approximations of the generalization error. Due to the relatively small size of the collected dataset, we conducted a 20-fold stratified cross-validation for both the proposed and baseline models to evaluate their prediction performance under this limitation better. The dataset was shuffled and split into a training set (950 samples) and a test set (50 samples) for each fold. We recorded the estimated item response parameters and predicted probabilities on the test set of each fold for further analysis. Since response prediction is a binary classification task, we used accuracy and F1 score as evaluation metrics. The average accuracies and average F1 scores of all folds on the training and test sets were reported. The F1 score, i.e., the harmonic mean of precision and recall, was used as a fairer metric than accuracy in an imbalanced dataset. Additionally, we conducted a Wilcoxon signed-rank test to determine if the cross-validation results of each fold between the proposed model and the baseline models were significantly different.

We also evaluated the prediction performance in the case of early prediction to see how much the performance changed when the prediction was made before the student answered the question. The general way of early prediction is fixed time-based, such as predicting the response at the first 12 seconds, as widely used in previous works (Joshi et al., 2019; Ruiz et al., 2021). In this research, we set a series of early prediction timings based on the quantiles of the response time distribution of the whole dataset. We truncated the feature-length according to the timing, only retaining the features no later than it. The early prediction timings included 12 seconds (the first quantile of the response time), 23 seconds (the median), 37 seconds (the third quantile), and 60 seconds (the full length of videos). We also reported the results for the most extreme case in which only the first video clip (approximately the first 1 second) was used for prediction.

To evaluate the interpretability of the proposed model, we compared the estimations of IRT parameters between the proposed model and Base IRT model in the previous study of Zhou et al. (2021). Base IRT model does not have student or item networks, and scalar parameters in the neural network estimate the student and item parameters. We observed a strong correlation between the estimated parameters of the Base IRT model and those inferred using the Markov chain Monte Carlo (MCMC) algorithm, which suggests that the model parameters can be interpreted as item-response parameters. To obtain the estimated IRT parameters, we iteratively trained Base IRT model using the entire dataset and kept the model that achieved the best prediction performance. The scalar parameters in the trained model represent the estimated IRT parameters. Additionally, we compared the estimations of SAD-IRT with Deep-IRT model, which has been shown to accurately estimate IRT parameters, especially for small datasets.

To evaluate whether the proposed model can be interpreted in the same way as IRT, we compared the similarities between the estimations made by the proposed model on test sets with Base IRT and Deep-IRT models. The root mean square error (RMSE), Pearson's correlation coefficient and the Kendall rank correlation coefficient were used as similarity metrics, following the work of Tsutsumi et al. (2021). Since the student and item networks estimated each student and item parameter multiple times on test sets, we used their averaged estimations for each student and item.

## 6 Results

### 6.1 Prediction performance

The overall dataset had a mean response value of 0.721 ± 0.449 (i.e., the mean value ± the standard deviation, SD), indicating that, on average, participants answered approximately 72.10% of the questions correctly. The average response time across all samples was 25.15 ± 15.44 seconds, ranging from 2.67 to 60.00 s. Base IRT model fitted the whole dataset and achieved 0.821 and 0.884 in terms of accuracy and F1 score.

The results of the cross-validation, including the average and SD of accuracies and F1 scores on the training and test sets, are shown in Tables 1, 2, respectively. Our proposed model, SAD-IRT, outperformed all baseline models, achieving average accuracies and F1 Scores of 0.857 ± 0.003 and 0.892 ± 0.006 on training sets, 0.844 ± 0.043 and 0.895 ± 0.028 on test sets, respectively. Overall, they were also the highest compared with all baseline models. Regarding the overall prediction performance on test sets for other baseline models, it was followed by Deep-IRT, Deep-IRT w/o BatchNorm, facial-video-only (MARLIN-TCN), and facial-video-only (OpenFace) in terms of accuracy and F1 score. The proposed SAD-IRT outperformed Deep-IRT, the second-best model, achieving 0.857 and 0.892 for averaged accuracy and F1 score on the training sets, compared to 0.832 and 0.878 for Deep-IRT, respectively, with *p* < 0.01 on a Wilcoxon signed-rank test. On the test sets, SAD-IRT achieved 0.844 and 0.895 for accuracy and F1 score, compared to 0.823 and 0.884 for Deep-IRT, respectively, with *p* < 0.05. We found that facial-video-only (MARLIN-TCN) outperformed facial-video-only (OpenFace) on the test sets, with a 0.9% and 0.4% increase in average accuracy and average F1 score, respectively. It also achieved higher results on the training sets, with a 0.1% and 1.3% increase. Comparing the results of Deep-IRT and Deep-IRT w/o BatchNorm models, we observed an overall performance improvement of 0.7% and 0.2% in terms of accuracy and F1 score for Deep-IRT model on the test sets. In addition, all the results of the evaluated models were at least higher than the chance level in the cross-validation.

**Table 1:** Performance of prediction on training sets using 20-fold cross-validation (mean ± SD).

**Model**	**Accuracies**	**F1 scores**
SAD-IRT (Proposed)	**0.857** ± 0.003 ^**^	**0.892** ± 0.006 ^**^
Deep-IRT (Tsutsumi et al., 2021)	*0.832* ± 0.003	0.878 ± 0.007
Deep-IRT w/o BatchNorm (Tsutsumi et al., 2021)	0.826 ± 0.003	*0.881* ± 0.004
MARLIN-TCN (Bai et al., 2018; Cai et al., 2023)	0.746 ± 0.002	0.838 ± 0.012
OpenFace (Joshi et al., 2019)	0.745 ± 0.004	0.825 ± 0.019

^**^p < 0.01. Bold values indicate the highest/best results. Italic values indicate the second-highest/second-best results.

**Table 2:** Performance of prediction on test sets using 20-fold cross-validation (mean ± SD).

**Model**	**Accuracies**	**F1 scores**
SAD-IRT (Proposed)	**0.844** ± 0.043 ^*^	**0.895** ± 0.028 ^*^
Deep-IRT (Tsutsumi et al., 2021)	*0.823* ± 0.042	*0.884* ± 0.026
Deep-IRT w/o BatchNorm (Tsutsumi et al., 2021)	0.816 ± 0.037	0.882 ± 0.023
MARLIN-TCN (Bai et al., 2018; Cai et al., 2023)	0.763 ± 0.033	0.857 ± 0.017
OpenFace (Joshi et al., 2019)	0.754 ± 0.021	0.853 ± 0.011

^*^p < 0.05. Bold values indicate the highest/best results. Italic values indicate the second-highest/second-best results.

**Figure 6:**
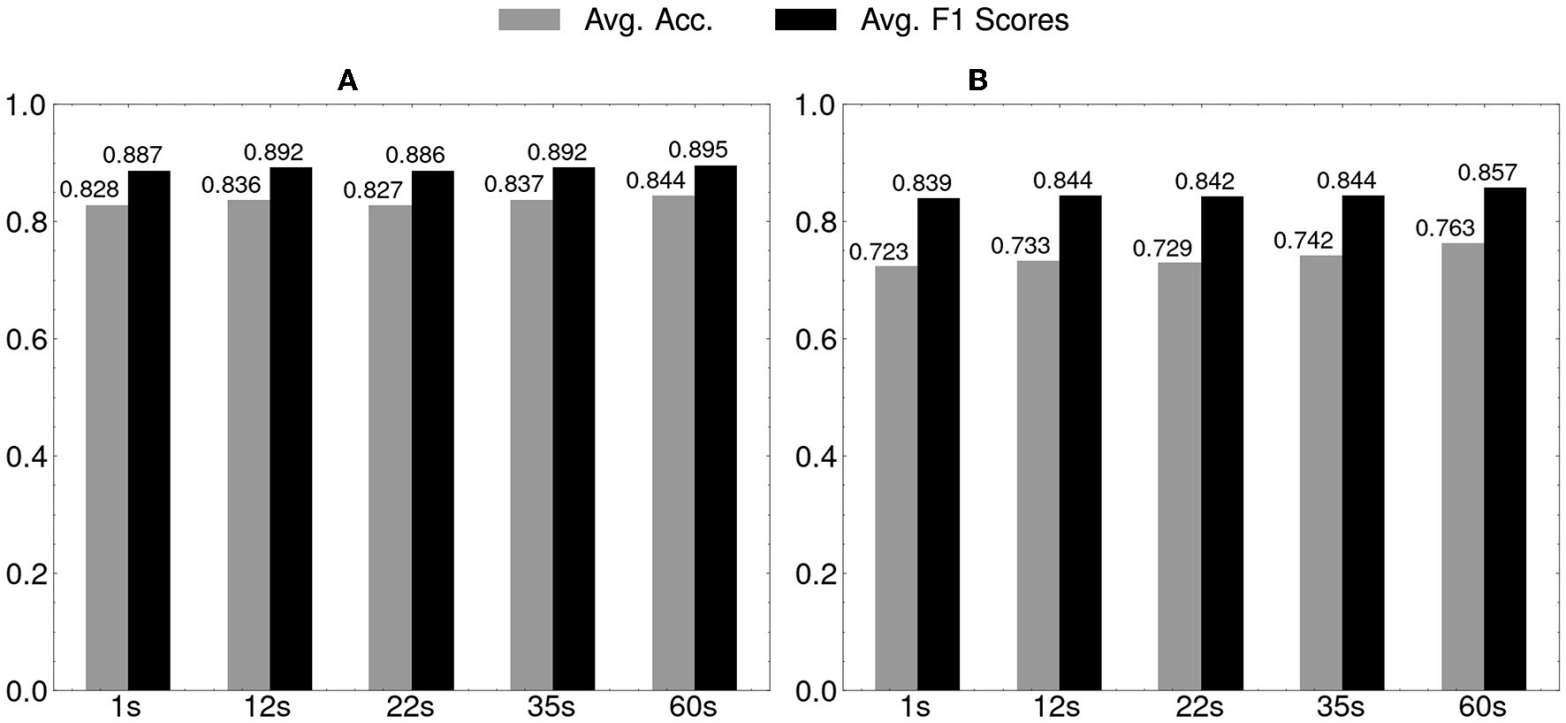
Early prediction results on test sets of 20-fold cross-validation: horizontal axis is for timings of early prediction. **(A)** SAD-IRT. **(B)** Facial-video-only (MARLIN-TCN).

Regarding the results of early prediction, the average accuracies and F1 scores of SAD-IRT and facial-video-only (MARLIN-TCN) on the test sets were presented in Figure 6, respectively. It was observed that in terms of accuracy and F1 score, the prediction performance of both models increased as they were given longer time to predict, ranging from the shortest 1 second to 60 seconds, which is the full length of the videos. The proposed model, SAD-IRT, still maintained better prediction performance than the student-state-unaware baseline model Deep-IRT which achieved the second-best prediction performance in the cross-validation. Even in the shortest 1s case, SAD-IRT was slightly better than Deep-IRT, with a 0.5% and 0.3% increase in accuracy and F1 score, respectively. As for MARLIN-TCN model, the overall prediction performance on the test sets decreased almost to the chance level in the shortest 1s case. Usually, the face was neutral at this moment after being presented with the fixation cross for 1s, and the student just began to read the question. Since facial muscle activities usually take hundreds of milliseconds to respond, as found in Dimberg and Thunberg (1998), the first-second features may only contain limited facial information.

### 6.2 Interpretability

**Figure 7:**
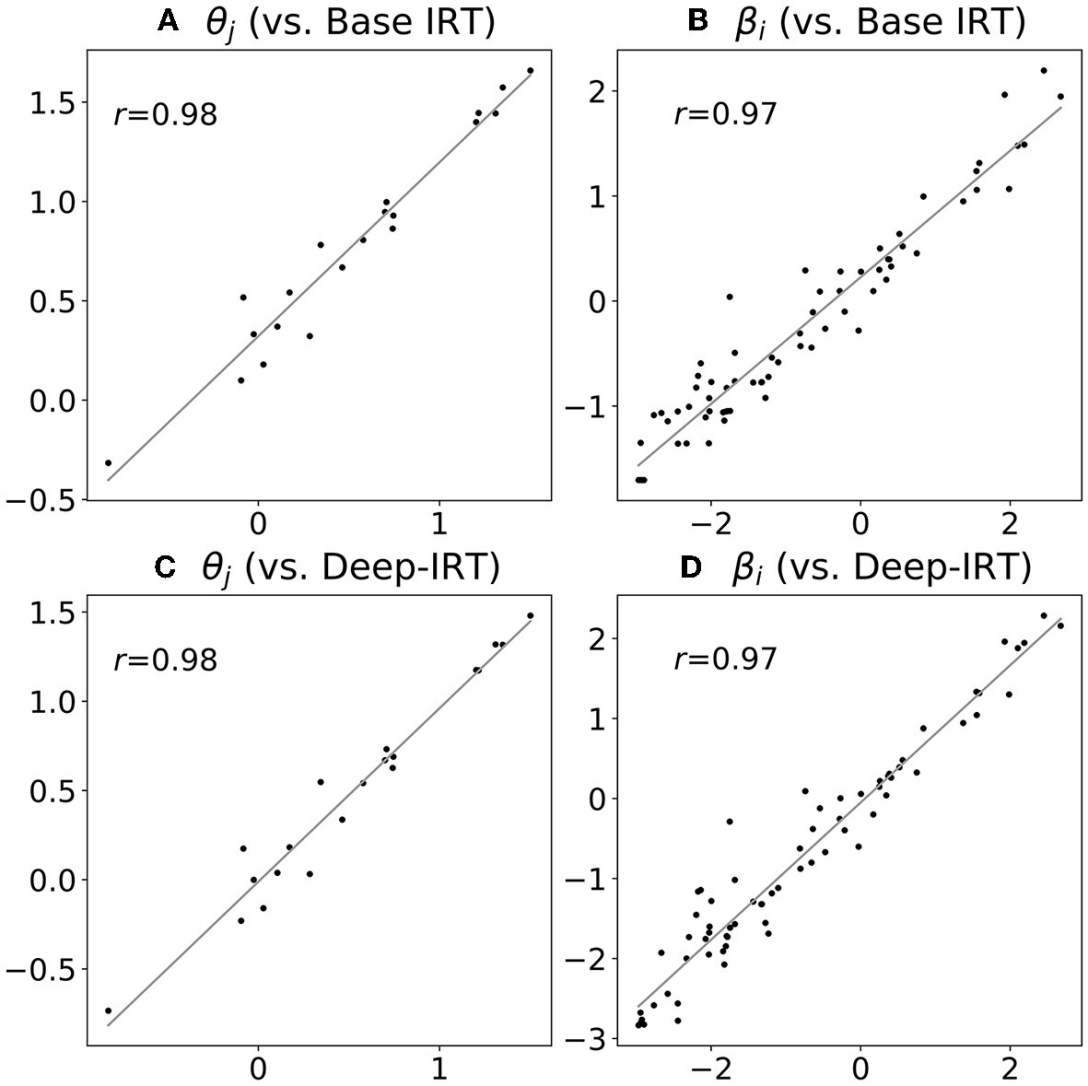
Estimated parameters: proposed model (horizontal axis) vs. base IRT (vertical axis). The number of dots in each plot equals the number of students for θ **(A, C)**, and the number of items for β **(B, D)**.

By comparing the estimated IRT parameters among the proposed model, Base IRT, and Deep-IRT, we found strong correlations (*r*≥0.97 and τ≥0.84) of estimated parameter values, including θ and β. Regarding RMSE, we found that the error of the student ability parameter θ was relatively small (RMSE was 0.29 and 0.12 for Base IRT and Deep-IRT, respectively) but not for the item difficulty parameter β (RMSE was 0.85 and 0.40), probably due to the larger sample size of items and the correlation with subjective difficulty ϕ. The details are summarized in Tables 3, 4, and also illustrated in the scatter plot in Figure 7. In summary, the proposed SAD-IRT model can estimate item-response parameters as accurately as IRT. In addition, since ϕ is our proposed parameter, it is difficult to compare it with IRT directly.

**Table 3:** Similarity of estimated IRT parameters between the proposed model (SAD-IRT) and Base IRT.

	**RMSE**	**Pearson (*r*)**	**Kendall (τ)**
Student ability θ	0.29	0.98	0.85
Item difficulty β	0.85	0.97	0.84

**Table 4:** Similarity of estimated IRT parameters between the proposed model (SAD-IRT) and Deep-IRT.

	**RMSE**	**Pearson (*r*)**	**Kendall (τ)**
Student ability θ	0.12	0.98	0.85
Item difficulty β	0.40	0.97	0.84

We calculated the statistics of student state estimations for SAD-IRT on the whole test sets. Regarding the ϕ parameter, we confirmed that the mean was 0.000 (while the SD was ± 0.781). In addition, the averaged student state ${\bar{\phi }}_{\cdot j}$ and ${\bar{\phi }}_{i\cdot }$ on each student and item showed weak negative correlations with θ (-0.157) and β (-0.276). The weak correlations between estimated item-response parameters and student states show they are independent.

We compared SAD-IRT's predictions on test sets with the student-state-unaware Deep-IRT's predictions and grouped them into two groups based on whether or not they made the same predictions. The number of samples in the group with the same predictions was 931, and the number of samples in the group with different predictions was 69, out of which SAD-IRT correctly predicted 45. Regarding the same predictions made by the two models, they achieved the same prediction performance. Only for those different predictions, either SAD-IRT model or Deep-IRT model correctly predicted, while the other failed. Obviously, SAD-IRT model outperformed Deep-IRT model because SAD-IRT made more correct predictions than Deep-IRT in the group of samples with different predictions.

We then compared the parameter estimations of SAD-IRT among groups. Regarding the group of the same predictions, the mean and SD of the item-response parameter and student state estimations were close to the estimations of all samples on test sets. However, we found great differences in the group of different predictions. Compared with the group of the same predictions, the mean student ability $\bar{\theta }$ was less proficient (0.370 ± 0.532 vs. 0.532 ± 0.614), and the item difficulty $\bar{\beta }$ was more difficult (0.039 ± 1.098 vs. -0.907 ± 1.555), and the student state $\bar{\phi }$ was much higher (0.591 ± 1.303 vs. -0.044 ± 0.710) as shown in Table 5.

**Table 5:** Statistics of estimated IRT parameters of SAD-IRT in different sample groups (mean ± SD).

**Samples**	**θ**	**β**	**ϕ**
All samples on test sets	0.521 ± 0.609	−0.842 ± 1.546	0.000 ± 0.781
Samples of the same predictions with Deep-IRT	0.532 ± 0.614	−0.907 ± 1.555	−0.044 ± 0.710
Samples of different predictions with Deep-IRT	0.370 ± 0.532	0.039 ± 1.098	0.591 ± 1.303

## 7 Discussion

The proposed model SAD-IRT demonstrated the best overall prediction performance compared to other baseline models. SAD-IRT significantly outperformed the second-best Deep-IRT model in the 20-fold cross-validation. The performance improvement was achieved by incorporating the student state into the IRT logistic function. SAD-IRT leverages facial video features to estimate student states and uses students' answers to estimate item response parameters. Additionally, we observed that Deep-IRT model with a deeper structure and Batch Normalization layers resulted in further improvements in prediction performance. MARLIN-TCN model trained on facial video representation features also resulted in higher prediction performance than OpenFace model trained on summary statistics of facial action unit features.

Regarding the results of the early prediction tasks shown in Figure 6, the proposed model, SAD-IRT, exhibited the ability to make predictions even before the student responded, surpassing the performance of the baseline models. This advancement is a good improvement compared to the results of the previous facial-video-only (OpenFace) method in early prediction (Joshi et al., 2019). The model's early prediction capability has a practical advantage in providing timely tutor instruction or intervention. Along with later features available, the general trend of the increase in SAD-IRT and MARLIN-TCN prediction performance validates that the facial video features contain crucial information for student performance prediction. Slight prediction performance improvement was achieved with very short and early facial features. In the shortest and earliest case of the early prediction at the first 1 second, it is clear that SAD-IRT achieved very similar results to Deep-IRT, while the result of MARLIN-TCN model almost degraded to the chance level.

By calculating SAD-IRT's parameter estimation mean and SD in the group of different predictions on test sets between SAD-IRT and the student-state-unaware Deep-IRT model, we found that SAD-IRT outperformed Deep-IRT on test sets, in the case of lower-skilled students ($\bar{\theta }$ = 0.370 for samples of different predictions group, compared to 0.521 for all samples) solving difficult tasks ($\bar{\beta }$ = 0.039 vs. -0.842), as shown in Table 5. Moreover, we observed that the mean value of the student state was higher as well ($\bar{\phi }$ = 0.591 vs. 0.000). These results indicate that when a low-skilled student attempts a difficult question, they perceive it as relatively more difficult, leading to an increase in ϕ. In this case, the predicted response probability decreases, and SAD-IRT predicted it to be wrong, unlike the student-state-unaware Deep-IRT model overestimating the response to be correct. This finding aligns with the relationship between student affect and ability as elaborated in Fenza et al. (2017), which suggests that if a question is too difficult, students may feel frustrated and anxious, ultimately causing an exit from the ideal learning, resulting in a failure to answer the question correctly. SAD-IRT can interpret the relative subjective difficulty through ϕ, particularly in the case of low-skilled students solving difficult tasks.

As a typical limitation in preliminary research, our study's small sample size of 20 students might limit its generalizability to a broader population. We thus performed additional analyses to validate the robustness of the proposed model (SAD-IRT) compared to Deep-IRT model, which is identical except for the exclusion of the key ϕ parameter. Our procedure involved leaving the data of one participant out of the 20-participant dataset (thus resulting in a dataset of 19 participants), and then conducting the same cross-validation with both SAD-IRT and Deep-IRT models. We repeated this process 20 times, excluding each participant once. The results from a paired t-test showed a significant performance improvement of SAD-IRT over Deep-IRT: for accuracy, t(19) = 13.11, p < 0.001, mean 0.899 ± 0.003 [SD] vs. 0.850 ± 0.004; and for F1 score, t(19) = 11.57, p < 0.001, 0.892 ± 0.002 vs. 0.838 ± 0.003. These results suggest that the participant size of 20 was acceptable for our evaluation study.

Another relevant concern is that cultural and ethnic biases in the application of facial features. However, focusing on a single culture and ethnicity, such as Japan, can be beneficial for ensuring diversity and representativeness within this scale. Japan is a homogenous and frequently explored culture/ethnicity, making it an ideal target for this study. Our participant selection of native Japanese university students was not gender-biased, with a female participation rate of 30% (6 out of 20 participants), comparable to the average student ratio at Japanese universities (30.8% for postgraduates and 44.5% for undergraduates^8^). Additionally, we found no significant gender differences in prediction performance in our test sets: for accuracy, t(998) = -0.872, p = 0.394, with means of 0.837 ± 0.049 [SD] for males and 0.860 ± 0.055 for females; for F1 score, t(998) = -0.763, p = 0.455, 0.885 ± 0.043 vs. 0.903 ± 0.053. Investigating other cultures and ethnicities is an important direction for future research. Delving into individual differences might require more explainable models, potentially at the expense of predictive performance (Rudin, 2019; Zhou et al., 2021), which is another intriguing topic but beyond the scope of the current study.

In this study, we focused on analyzing student state within one problem-solving, potentially overlooking student states carried from one item to another. To investigate whether there is any temporal dependence among student states, we explored how the student state ϕ parameter evolved by applying a first-order autoregressive AR(1) model to each participant's ϕ values. This analysis revealed no temporal trend, either increasing or decreasing. The autoregressive coefficient for each participant was not significantly different from zero (p = 0.51 [mean] ± 0.28 [SD], with a minimum of 0.07 and a maximum of 0.96). This lack of trend could be partially attributed to the video segment for each problem-solving. More naturalistic scenarios without such segmentation might reveal a stronger temporal structure. Temporal trends may be also observable in different contexts, such as in tests with a predominance of either easier or more difficult items, which could induce emotions like boredom. In such scenarios, it would be beneficial to integrate attention networks, as proposed in Tsutsumi et al. (2022); our ϕ parameter will be constrained by its previous estimations as well.

Regarding the interpretability of the IRT parameters, we found that the proposed model, SAD-IRT, accurately estimated the IRT parameters, which was evident from the high Pearson and Kendall correlation coefficients observed among SAD-IRT, Base IRT, and Deep-IRT. The results demonstrated that the parameter estimations were highly similar, as shown in Figure 7. Additionally, since the mean of ϕ was zero, the student and item parameters were less biased, with ϕ included in SAD-IRT. Given the high similarities between the item-response parameter estimations of SAD-IRT and the base models, SAD-IRT can interpret the estimated IRT parameters, such as the student ability parameter θ and the item difficulty parameter β, in the same way as IRT.

The relationship between emotion and student performance, similar to the ϕ-emotion relationship, is also complex due to the diverse emotions present in the learning domain (Pekrun, 2011; Fenza et al., 2017). Furthermore, we had to consider the trade-off between predictive performance and model interpretability (Rudin, 2019). Given these factors, we found establishing a clear relationship challenging. Therefore, in this paper, we focused on extracting the most effective features to directly regress our relative subjective difficulty parameter, ϕ, instead of identifying emotions, to maintain prediction performance. To explore the ϕ-emotion relationship within our model's framework, we recognize at least two potential methods. One involves employing video-based facial emotion recognizers, as those in Goncalves et al. (2023), for a post hoc analysis, although such analyses are sometimes not advisable (Zhou et al., 2021). Alternatively, another classifier layer could be added to our model to process the extracted facial spatiotemporal features. While these approaches exceed the scope of this paper, they offer promising future research directions, particularly if ground truth emotion labels are available. Such methods could significantly improve the interpretability of the ϕ parameter.

Overall, the proposed SAD-IRT model outperformed the baseline models regarding overall prediction performance and maintained its performance in early prediction tasks. By incorporating the student state into IRT using facial features, SAD-IRT model demonstrated the best prediction performance and enhanced the interpretability of the model by introducing the relative subjective difficulty parameter, i.e., the student state ϕ. SAD-IRT is useful in helping instructors understand students' learning experiences through accurate student performance prediction and the interpretability of the relative subjective difficulty of the current task.

Furthermore, in addition to student performance prediction, SAD-IRT model has the potential to predict students' dropout behaviors in the future. High dropout rates are a major challenge in online learning (Lee and Choi, 2011). As facial expressions may be elicited when dropout behaviors occur, potentially due to difficult tasks, SAD-IRT can predict dropouts similarly by capturing the student state from facial features and estimating item-response parameters. The results are also interpretable to stakeholders and can help prevent dropouts.

## 8 Conclusion

In conclusion, this research proposed the SAD-IRT model for predicting student responses using facial videos and response data. The SAD-IRT model incorporated the student state parameter into (Deep-)IRT using facial videos, resulting in superior overall prediction performance compared to baseline models. The model also maintained its performance in early predictions, surpassing the previous facial-video-only model. The accurate estimation of IRT parameters and the student state enhances the interpretability of the model in educational applications, allowing for appropriate learning interventions based on student ability and subjective difficulty. Additionally, the SAD-IRT model has the potential to predict dropout behaviors, providing valuable insights for preventing dropouts in online learning settings.

## Data availability statement

The datasets presented in this article are not readily available because the dataset is only partially available for response data and high-level facial video features (OpenFace). However, some high dimensional data of facial features (such as those extracted by the MARLIN model) are not appropriate to open publicly. Requests to access the datasets should be directed to yan@ai.iit.tsukuba.ac.jp.

## Ethics statement

The studies involving humans were approved by University of Tsukuba, Institute of Systems and Information Engineering, Internal Ethics Review Board (Approval Number: 2022R664). The studies were conducted in accordance with the local legislation and institutional requirements. The participants provided their written informed consent to participate in this study.

## Author contributions

YZ: Conceptualization, Data curation, Formal analysis, Methodology, Software, Validation, Visualization, Writing – original draft, Writing – review & editing, Investigation. KS: Conceptualization, Funding acquisition, Project administration, Resources, Supervision, Writing – review & editing. SK: Conceptualization, Project administration, Resources, Supervision, Writing – review & editing, Methodology.

## Footnotes

^1^https://sites.google.com/site/assistmentsdata/home/2009-2010-assistment-data?authuser=0, [Accessed: 27-Aug-2023].

^2^Wikipedia. SPI . https://ja.wikipedia.org/wiki/SPI  [Accessed 22-August-2023].

^3^https://www.3anet.co.jp/np/secure/0-0001-03-401800/0-0001-03-401800-1.pdf, [Accessed: 29-Nov-2023].

^4^オフィス海(2021). 史上最強. SPI & テストセンター超実戦問題集 2021 最新版. Tokyo:ナツメ社.

^5^SPIノートの会(2020). これが本当のSPI3だ! 2020. 年度版Tokyo:洋泉社.

^6^ノマド・ワークス(2017)本気で内定! SPI & テストセンター -1 2 0 0  (2017年度版). Tokyo:新星出版社.

^7^Technically, the MARLIN model extracts spatial-temporal features from a short video clip segmented from the input video. Here, we still refer to it as the spatial feature extractor for simplicity, while the latter one, the temporal feature extractor, learns temporal features over the whole video's features instead.

^8^https://www.mext.go.jp/en/news/topics/detail/__icsFiles/afieldfile/2017/10/13/1397166_001_1.pdf, [Accessed: 29-Nov-2023].
